# Status Epilepticus in a Tertiary Care Hospital in Morocco: A Retrospective Analysis

**DOI:** 10.7759/cureus.50591

**Published:** 2023-12-15

**Authors:** Ibrahim Bechri, Abdelkrarim Shimi, Ali Derkaoui, Mohammed Khatouf

**Affiliations:** 1 Anesthesiology and Intensive Care Department, Hassan II University Hospital, Fez, MAR

**Keywords:** treatment, risk factors, electroencephalogram, refractory status epilepticus, status epilepticus

## Abstract

Background

Status epilepticus (SE) is a common neurologic emergency with high rates of mortality and morbidity.

Objective

To analyze the clinical characteristics, causes, management, and outcomes of patients with SE in a tertiary care hospital in Morocco.

Methods

A retrospective study was conducted from January 2019 to December 2021, including all patients admitted to the medico-surgical general intensive care unit (ICU) with a diagnosis of SE. We recorded demographic characteristics, SE clinical history, management, causes, and discharge outcomes.

Results

Overall, 82 patients with SE were included, the median age was 39.5 years (18-95), 61% of the patients were male, the majority of semiology was convulsive SE (93%, N: 77), epilepsy of unknown cause was the most common diagnosis (41.2%, N: 34), and the most known etiology was acute/subacute cerebrovascular events (12 patients, 14.4%). All patients received benzodiazepines, 96.4% of them received phenobarbital as a second line of treatment, 65 patients required anesthesia, 52 patients developed one complication at least - the most common complication being systemic infection, and the mortality rate was noted to be 38% among patients with SE (N: 31). In this study, the factors associated with mortality were ischemic stroke (as an etiology of SE (p=0.048), history of epilepsy (p=0.005), poor therapeutic adherence (p=0.001), cardiovascular complications, presence of multiple complications (p=0.0001), pneumonia (p=0.0001), and the recurrence of SE (p=0.050).

Conclusions

We provide a single-center retrospective analysis of admissions in SE and note that mortality among SE patients is high in our settings. Improving prehospital emergency care and implementing elective ICU admission for patients at high risk could improve the mortality rate.

## Introduction

Status epilepticus (SE) occurs within five minutes or more of continuous clinical and/or electrographic seizure activity or recurrent seizure activity without consciousness recovery between seizures. It is a common, life-threatening neurologic disorder with high morbidity and mortality rates [1]. Its consequences can result in alterations of the neuronal network or death, and its incidence ranges from 10 to 40 cases per 100,000, with the rate of mortality ranging from 7.6 to 39% [2]. Rapid management is a priority to improve patient outcomes while etiologic investigation can be a challenge in people with SE. In Morocco, most people with epilepsy have no access to treatment, with traditional customs leading to very late patient management. Data on SE are lacking, and recognizing the burden of SE morbidity, we conducted a single-center retrospective study. The objective was to analyze the clinical characteristics of SE and its etiology. We also report mortality rates and risk factors.

## Materials and methods

Study design

We conducted a single-center retrospective study at the A1 intensive care unit at the Hassan II University Hospital of Fez in Morocco. Three-year study data were obtained from January 2019 to December 2021. We included patients with SE according to defined inclusion and exclusion criteria.

Inclusion and exclusion criteria for the study population

Adult patients ( age > 17 years) admitted to the intensive care unit with the diagnosis of SE were included in the study and data were obtained from medical records. Pregnant and lactating women, children, and patients with incomplete clinical data were excluded.

Definition of variables

All pre- and in-hospital records of SE patients were reviewed and data were collected using standardized forms. SE was defined according to the latest criteria of the International League Against Epilepsy (ILAE). SE involves a convulsive seizure lasting 5 minutes or more, nonconvulsive status with impaired consciousness lasting longer than 10 minutes, while refractory status epilepticus (RSE) is defined as persisting seizures after the failure of a sufficient benzodiazepine dose as a first-line treatment and an antiseizure medication (ASM) as a second-line treatment, irrespective of time [3].

Baseline variables

These included gender, age, family history of epilepsy, SE type, SE duration, symptomatology, electroencephalogram (EEG), neuroimaging, cerebrospinal fluid results, etiology of seizure treatment, complications, length of hospital stay, and outcome.

SE duration

The end of SE was defined clinically as a cessation of seizure activity on EEG for patients with nonconvulsive status or under pharmacological sedation [3], continuous EEG monitoring was not available, and an EEG examination was performed to confirm the cessation of SE.

Neuroimaging

This was performed in all cases on admission and was repeated according to clinical evolution.

Etiologies of seizures

The etiology was rated as unknown if no cause of SE could be identified.

Management of SE

According to our hospital’s management protocol, anti-seizure medication (ASM) was administered per the national guidelines, with the administration of benzodiazepines in the initial phase (diazepam or midazolam), followed by intravenous phenobarbital as a second line of treatment if SE did not resolve. Patients with refractory SE required sedation.

Statistical analysis

Data were analyzed using SPSS Statistics software version 22.0 (IBM Corp., Armonk, NY, USA). Frequencies were calculated using descriptive statistics, mean with standard deviation was used for continuous variables, and univariate comparisons of proportions were calculated using a chi-square test. P values <0 .05 were considered statistically significant. Predictive factors of mortality were studied with a logistic regression model with multivariate analysis.

## Results

Overall, 82 patients with SE were admitted to the ICU from January 2019 to December 2021 and were included in the study.

Patient characteristics

Patients were aged 18 to 95 years, with a mean of 39.5 years, including 50 males (61%) and 32 females (39%). Seventy-two percent (72%) of patients (N: 59) presented with de-novo SE, 27.7% of patients (N: 23) had a history of epilepsy, of whom 40% (N: 9) were receiving regular antiepileptic drugs (AED) and 60% (N: 14) had poor therapeutic adherence. Sixteen patients (18%) had a history of brain injury, the majority of semiology was convulsive SE (93%, N: 77); and 7 patients had non-convulsive SE. EEG was only performed in 45 patients (54%). All patients underwent head CT scans, which showed abnormalities in 45 patients. MRI was performed on 48 patients, which showed abnormalities in 37 cases. Lumbar puncture was performed in 65 cases, and cerebrospinal fluid culture tested positive in 5 cases. Metabolic abnormalities notified at admission were hypernatremia, hyponatremia, and kidney failure.

Causes of SE

**Figure 1:**
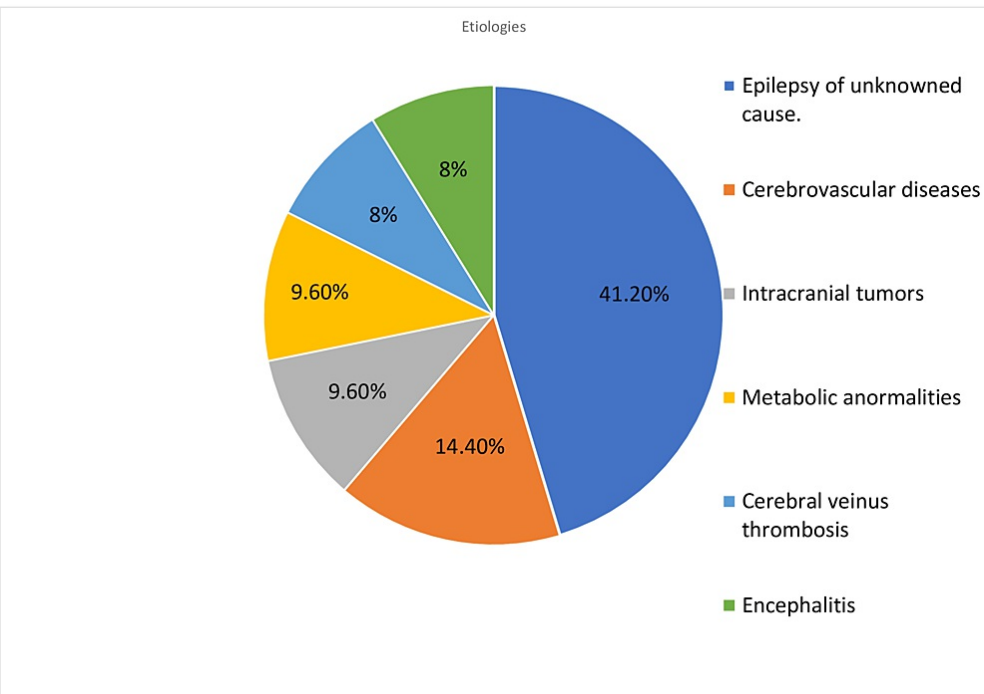
Proportion of status epilepticus etiologies

Epilepsy of unknown cause was the most common diagnosis (41.2%, N: 34). The cause of SE in 19 patients was a reduced seizure threshold. The most known etiology was acute/subacute cerebrovascular events (12 patients, 14.4%), primary tumors of the CNS (8 patients, 9.6%), metabolic abnormalities (8 patients, 9.6%), cerebral venous thrombosis (7 patients, 8%), and encephalitis (7 patients, 8%). The range of etiologies of super-refractory status epilepticus (SRSE) is illustrated in Figure 1.

Drugs used to control SE

All patients received benzodiazepines (diazepam or midazolam) as the first line of treatment, 96.4% of them received phenobarbital as the second line of treatment, and 65 patients had refractory SE requiring anesthesia.

Patient characteristics are listed in Table 1.

**Table 1:** Patient characteristics SE: status epilepticus

Items	Cases	Ratio
Sex (Male/Female)	50/32	
History of epilepsy	23	27.7%
Semiology		
Convulsive SE	77	93%
Non convulsive SE	5	6%
Etiology		
Unknown	12	14.4%
Vascular	8	9.6%
Tumoral	8	9.6%
Metabolic	6	8%
Infectious	6	8%
Cerebral venous thrombosis	6	8%
Refractory SE	65	79%
Acute complication	52	63%
Mortality	31	38%

Outcomes

Fifty-two patients developed at least one complication, the most common was a bloodstream infection, 31 patients (38%) died, mortality was attributed to cardiovascular and infectious complications in most cases, and the median days to mortality was 15.23 days.

Mortality risk factors

Acute Physiology and Chronic Health Evaluation (APACHE) II score ≥10 (p=0.0001), ischemic stroke (as an etiology of SE (80% vs 66.2%, P=0.048)), history of epilepsy(93% vs 66%, P =0.005), poor therapeutic adherence (100% vs 72%, P=0.001), cardiovascular complications (90% vs 43%, P= 0.0001), and presence of multiple complications (P=0.0001), pneumonia (96.7% vs 11.7%, P=0.0001), recurrence of SE (70.9% vs 58%, P=0.050) were variables significantly associated with mortality. However, there were no significant differences in mortality in relationship with the type of SE (for convulsive SE, P=0.419; refractory SE (P=0.173); or tracheal intubation, P=0.321 (Table 2).

**Table 2:** Risk factors related to mortality SE: status epilepticus

	Death (N =31 ) 38%	Recovery (N = 51) 62%	P
Ischemic stroke	25 (80%)	49 (66.2%)	0.048
History of epilepsy	29 (93%)	34 (66%)	0.005
Poor therapeutic adherence	31 (100%)	37 (72%)	0.001
Cardiovascular complication	28 (90%)	22 (43%)	0.0001
Pneumonia	30 (69.7%)	6 (11.7%)	0.0001
Recurrence of SE	22 (70.9%)	30 (58%)	0.05

## Discussion

Our study analyzed the clinical characteristics, etiologies, outcomes, and factors associated with mortality in patients with SE in a Moroccan center.

Demographic and clinical characteristics

The median age was 39.5 years, the incidence of SE was higher in elderly people in several studies, and 61% of patients were male, which is consistent with previous studies [4-5]. The rate of patients with a history of epilepsy in SE is up 40% to 50% in several studies [6-7]. Only 27.7% of our patients had a history of epilepsy, illustrating the high rate of de-novo SE in our study, with the possibility of misdiagnosed epilepsy remaining abnormally high in our region [8]. Most SE was convulsive (93%, N: 76) in our study; only 7% of SE was non-convulsive, in consistency with other series [4,5,7]. Convulsive SE is usually easy to diagnose and EEG is not required for the initial diagnosis; however, the possibility of progressing to pauci-symptomatic epilepsy justifies daily EEG monitoring until recovery of consciousness or refractory SE [8-9]. In our context, EEG monitoring access is limited, leading to an underestimated incidence of nonconvulsive SE and an increase in the duration of sedation in refractory SE.

Causes of SE

In our study, we found a higher rate of unknown etiology (41.2%), considering that MRI was not always accessible in our context, particularly for unstable patients, followed by patients with acute symptomatic etiology; cerebrovascular events were found to be the main known cause (14.4%) in conformance with several studies [10-11]. It is worth mentioning that, recently, autoimmune/paraneoplastic encephalitis has become one of the most known causes of SE [12-13], probably undiagnosed and untreated in our study because anti-neuronal autoantibodies testing is not yet available nationally.

Treatment

Our study indicated that the use of benzodiazepines accounted for 96.4%, with phenobarbital as the second line to control seizure activity. Intravenous levetiracetam, phenytoin, and valproic acid recommended for second-line treatment are not available in our country. Sixty-five patients (79%) required anesthesia to be maintained for at least one to two days to have burst suppression. Midazolam and propofol were the main anesthetic drugs; a recent international cohort study proved that propofol and midazolam are equivalently efficacious for refractory SE [14]. We reserved ketamine in association with other anesthetics for SRSE. Publications on ketamine efficiency in SE are heterogeneous, but ketamine appears to have a promising outlook for refractory SE and SRSE. Larger randomized prospective studies should clarify its place in controlling seizures [15].

Outcomes

In contrast to several studies of SE that found mortality rates of approximately 10-20%, the outcome of patients with SE in our study is poor with 38% mortality; this could be explained by the lack of prehospital emergency care and specialized centers and limited access to EEG monitoring as well as limited diagnostic facility limitations. Several studies have shown that delay in treatment and a longer duration of SE contributed to poor clinical outcomes [16-17].

Mortality risk factors

A history of epilepsy, ischemic stroke as an etiology, poor therapeutic adherence, the presence of complications, and recurrence of SE were associated with poor prognosis. This is compatible with the findings of other researchers, which identify also the age of the patient and duration of SE as risk factors for mortality [18-20]. Also, studies have shown that in patients with ischemic stroke or those suffering from an anoxic brain injury, the occurrence of SE is identified as an independent factor of mortality [21-22]. Immediate admission to the ICU of patients with a high risk of mortality should improve the prognosis of these patients.

## Conclusions

This study elucidated the clinical characteristics, etiologies, management, and outcomes of SE in our hospital. The patients in our study were young, with a high rate of de-novo SE. While cerebrovascular events are the most common cause of known etiology, the major diagnosis in our study was unknown etiology due in part to autoimmune encephalitis, most likely undiagnosed. The majority of SE patients in our study were managed with benzodiazepines and phenobarbital; patients who required anesthesia received midazolam or propofol. The mortality rate in patients with SE remained high in our study. Rapid determination of the causative etiology and initiation of therapy could decrease the mortality rate by improving prehospital emergency care and implementing elective ICU admission for patients at high risk. In our study, a history of epilepsy, ischemic stroke as an etiology, poor therapeutic adherence, presence of complications, and recurrence of SE were associated with a poor prognosis.
